# Ruptured Splenic Artery Aneurysm: Rare Cause of Shock Diagnosed with Bedside Ultrasound

**DOI:** 10.5811/westjem.2015.7.25934

**Published:** 2015-10-20

**Authors:** Terri Davis, Joseph Minardi, Jennifer Knight, Hollynn Larrabee, Gregory Schaefer

**Affiliations:** * West Virginia University School of Medicine, Morgantown, West Virginia; † West Virginia University, Department of Emergency Medicine, Morgantown, West Virginia; ‡ West Virginia University, Department of Surgery, Morgantown, West Virginia

## Abstract

Splenic artery aneurysm rupture is rare and potentially fatal. It has largely been reported in pregnant patients and typically not diagnosed until laparotomy. This case reports a constellation of clinical and sonographic findings that may lead clinicians to rapidly diagnose ruptured splenic artery aneurysm at the bedside. We also propose a rapid, but systematic sonographic approach to patients with atraumatic hemoperitoneum causing shock. It is yet another demonstration of the utility of bedside ultrasound in critically ill patients, specifically with undifferentiated shock.

## INTRODUCTION

Ruptured splenic artery aneurysm (SAA) is a rare condition that is challenging to diagnose given the nonspecific presentation. Non-specific abdominal pain is common in the emergency department (ED) representing 4–5% of complaints.^1^ The incidence of SAAs is low, seen incidentally in only 0.78% of patients undergoing angiography.^2^ Of these, only about 10% will rupture.^3^ We report a case of splenic artery aneurysm rupture that emphasizes the value of ultrasound performed in the ED in shortening the differential, decreasing time to diagnosis, and altering the management plan with benefits in patient outcome. This study did not need to be approved by our university’s institutional review board, as case studies are not considered by our institution to be “human subjects’ research.”

## CASE REPORT

A 41-year-old woman presented to the ED with sharp, stabbing chest pain radiating into the abdomen and the back with nausea and diaphoresis. She reported diffuse abdominal pain for several months, and admitted to only occasional alcohol use. Cholecystectomy was her only surgical history.

Initial vital signs were BP 82/60 and pulse 110. Physical examination showed a diffusely tender abdomen with increased pain in the left upper quadrant and epigastric regions. Vital signs improved initially with an intravenous (IV) fluid bolus.

The initial differential included upper gastrointestinal bleeding, sepsis, myocardial infarction, aortic emergencies, pregnancy complications including ectopic, and perforated viscus.

Chest radiograph and electrocardiogram were normal. Despite initial stabilization, the patient again became hypotensive with signs of profound shock including an ashen appearance, decreased mental status, and weak, thready pulses.

**Figure 1:**
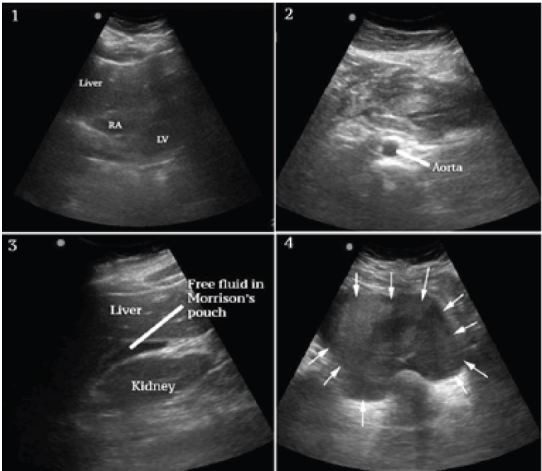
Frame 1 shows a subxiphoid view of the heart without pericardial effusion or RV dilation. Additonally, left ventricle (LV) function was vigorous. RA-right atrium. Frame 2 shows a portion of the abdominal aorta with a normal diameter. Frame 3 shows free fluid in Morison’s pouch. Frame 4 shows fluid with mixed and increased echogenicity in the pelvis consistent with blood (arrows).

**Figure 2:**
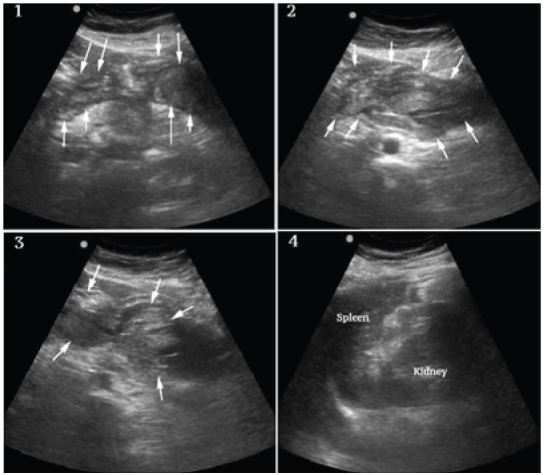
Frames 1–3 show alternate views of the extensive and organized clot formation in the epigastrium and left upper quadrant (arrows). Frame 4 shows the spleen which is grossly normal in size and appearance.

A bedside ultrasound was performed to evaluate the patient’s physiology and potential etiology of shock. The cardiac views were limited but showed no effusion or obvious right ventricular dilation, and left ventricular function appeared vigorous (Figure 1, Frame 1). The visualized portions of the abdominal aorta were of normal caliber as seen in Figure 1, Frame 2. Extensive free peritoneal fluid with areas of increased and mixed echogenicity was noted in Morison’s pouch (Figure 1, Frame 3, and Video), the paracolic gutters and pelvis (Figure 1, Frame 4, and Video). There was extensive clot formation in the epigastrium and left upper quadrant (Figure 2, Frames 1–3) but not surrounding the spleen, which appeared normal (Figure 2, Frame 4). There were no obvious adnexal masses (Figures not available due to technical machine storage malfunction) and the previously ordered human chorionic gonadotropin (HCG) had returned negative.

At this point the differential was modified and included spontaneous splenic rupture, but from previous clinician experience, this was felt less likely due to the normal appearance of the spleen on ultrasound. Hemorrhagic pancreatitis was considered, but the extent of intraperitoneal hemorrhage and clinical presentation did not appear consistent. Ruptured ectopic pregnancy and hemorrhagic ovarian cyst were also felt unlikely given the lack of adnexal mass and negative HCG. SAA was felt the most likely diagnosis given the overall clinical and sonographic findings, specifically diffuse atraumatic hemoperitoneum, the localized clot formation in the epigastrium and left upper quadrant and lack of findings to support other differential considerations.

Adequate IV access was assured and resuscitation with blood was initiated while the patient was taken immediately to radiology for computed tomography (CT) angiography, which showed multiple SAAs and ongoing hemorrhage. Interventional radiology and surgery were consulted. The patient was taken to a dual angiography/operating room suite where splenic artery embolization was performed, followed by open evacuation of hematoma, splenectomy, distal pancreatectomy and further hemorrhage control. Resuscitation followed a massive transfusion protocol, resulting in total administration of seven units of packed red blood cells, four units of fresh frozen plasma, one unit each of platelets and cryoprecipitate, in addition to autotransfusion during surgery. She did well postoperatively.

## DISCUSSION

Ruptured SAAs are an uncommon cause of hemorrhagic shock but the splenic artery accounts for 60% of visceral aneurysms.^2^ SAAs have a 4:1 female to male ratio statistically related to multiparity with a mean of 3.5 pregnancies.^2^ This is believed to be related to hormonal influences and increased splenic arterial wall stress from portal hypertension during pregnancy. Portal hypertension from other causes is also believed to be a contributing factor.^2^ Our patient had no known risk factors for a ruptured SAA other than her female gender, making her low probability for this diagnosis.

After rupture, SAAs cause significant blood loss with hemodynamic instability typically occurring in 6–96 hours, giving time for repair if diagnosed. The mortality ranges from 10–36% in non-pregnant patients^3^–^4^ but doubles for pregnant patients and those with pre-existing portal hypertension.^4^ Rapid diagnosis and intervention are critical.

Initial presentation of rupture is chest pain followed by hemodynamic instability 6–96 hours later. The delayed blood loss is caused by the “double rupture phenomenon,” where blood is initially contained within the lesser omental sac, delaying the onset of intraperitoneal hemorrhage.^5^ This provides a window for diagnosis and treatment that may reduce the current mortality rate.

Ruptured SAA is most frequently reported in pregnancy. Only a few reported cases described the use of bedside ultrasound to identify hemoperitoneum prior to open laparotomy. Jackson et al.^4^ described two cases of females with hemodynamic collapse: one in a patient at 35-weeks gestation and another in a woman with signs of shock and a suspected obstetric etiology. Grousolles et al.,^5^ report a woman at 6-weeks gestation presenting with signs of shock and an initial suspected diagnosis of ruptured ectopic pregnancy. Heitkamp et al.^6^ report a woman at 31-weeks gestation complaining of sudden severe abdominal pain and hypotension, with hemoperitoneum on ultrasound, who underwent laparotomy where a suspected ruptured SAA was identified and surgically treated.

The diagnosis of SAA primarily occurs when a CT with contrast is ordered as part of the work up of abdominal pain or during exploratory surgery for non-traumatic hemoperitoneum.

Etiologies of non-traumatic hemoperitoneum with hemodynamic instability include ruptured vascular neoplasm in a solid organ, spontaneous splenic rupture, ruptured ectopic pregnancy, uterine rupture during pregnancy, uterine artery rupture, or intraperitoneal abdominal aortic aneurysm rupture. A ruptured hemorrhagic ovarian cyst may cause hemoperitoneum, but hypotension is atypical.^7^ When SAA occurs during pregnancy, 70% are initially diagnosed as uterine ruptures.^8^

When using ultrasound to assess cases of non-traumatic shock with hemoperitoneum, a careful consideration of the differential diagnosis with a rapid but systematic sonographic evaluation may suggest the most likely etiology. In this case, the absence of clot or fluid around the spleen implied spontaneous spleen rupture was unlikely. This belief was based mostly on clinician experience, but there have also been reports of spontaneous spleen rupture that report splenomegaly, perisplenic hematoma and/or fluid collections as common sonographic findings.^9^ The absence of adnexal masses and negative HCG made ectopic or other adnexal etiologies seem unlikely. The normal diameter of the aorta made intraperitoneal abdominal aortic rupture unlikely. Other uterine pathology was felt unlikely given the grossly normal size of the uterus and the fact that these are typically complications of later pregnancy. Lastly, the localized, extensive clot formation in the epigastrium and left upper quadrant strongly suggested a ruptured SAA. Additional analysis with color and Doppler modalities could be considered for similar cases, but were not performed in this case. Preliminary diagnosis made using a modified rapid ultrasound in shock^10^ protocol in patients with hemodynamic instability correlates strongly with final diagnoses,^11^ suggesting ultrasound has potential in guiding first-line therapeutic approach as it did in this case.

## CONCLUSION

We report a patient who presented with nonspecific complaints and undifferentiated hypotension where bedside ultrasound assisted in drastically altering the differential. Identifying the rare diagnosis of ruptured splenic artery aneurysm early led to rapid intervention and a more favorable outcome for the patient. This case further illustrates the utility of bedside ultrasound in the evaluation of critically ill patients, specifically in undifferentiated shock. We suggest a rapid but systematic sonographic evaluation to assist in determining the etiology of nontraumatic hemoperitoneum causing shock. The absence of sonographic signs of other etiologies combined with the finding of extensive clot formation in the epigastrium and left upper quadrant may suggest ruptured splenic artery aneurysm earlier in the patient’s course, expediting diagnosis and management, and potentially improving outcome.

## Figures and Tables

**Video:** Narrated overview of the key findings and video clips. Free peritoneal fluid and intraperitoneal clot is shown as well as a normal appearing spleen.
